# Supplementary material for: High-throughput transposon mutagenesis in the family Enterobacteriaceae reveals core essential genes and rapid turnover of essentiality
Source: mBio. 2024 Aug 29;15(10):e01798-24. doi: 10.1128/mbio.01798-24 (PMC11481867; doi:10.1128/mbio.01798-24)
Supplement: Supplemental material — Figures S1-S6 and Table S1. [file mbio.01798-24-s0001.pdf]

SUPPLEMENTARY FIGURES & TABLES

High-throughput transposon mutagenesis in the family Enterobacteriaceae reveals core essential genes and rapid turnover of essentiality

Fatemeh A. Ghomi, Jakob J. Jung, Gemma C. Langridge, Amy K. Cain, Christine Boinett, Moataz Abd El Ghany, Derek J. Pickard, Robert A. Kingsley, Nicholas R. Thomson, Julian Parkhill, Paul P. Gardner, Lars Barquist

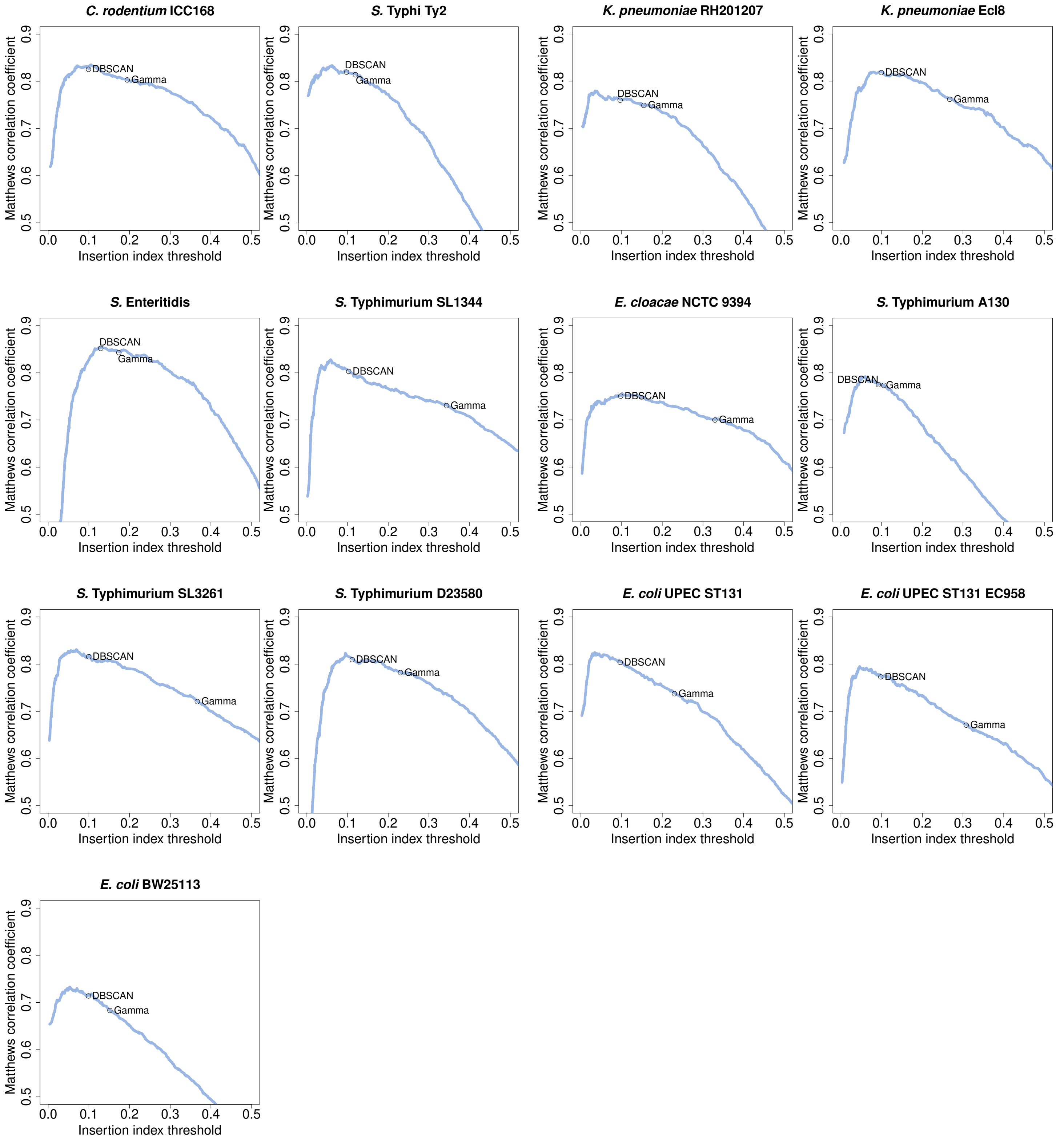

Figure S1: Matthew's correlation coefficient calculated across insertion index thresholds for all 13 bacteria in this study. True positives are genes predicted as essential at a given cut-off whose orthologs are classified as essential in *E. coli* K-12 by the EcoGene database. False positives are genes predicted as essential at a given cut-off, but whose orthologs are not classified as essential by EcoGene. The cut-offs derived from DBSCAN clustering and fitted gamma distributions are shown in each figure. DBSCAN-derived cut-offs outperform gamma fits in all cases.

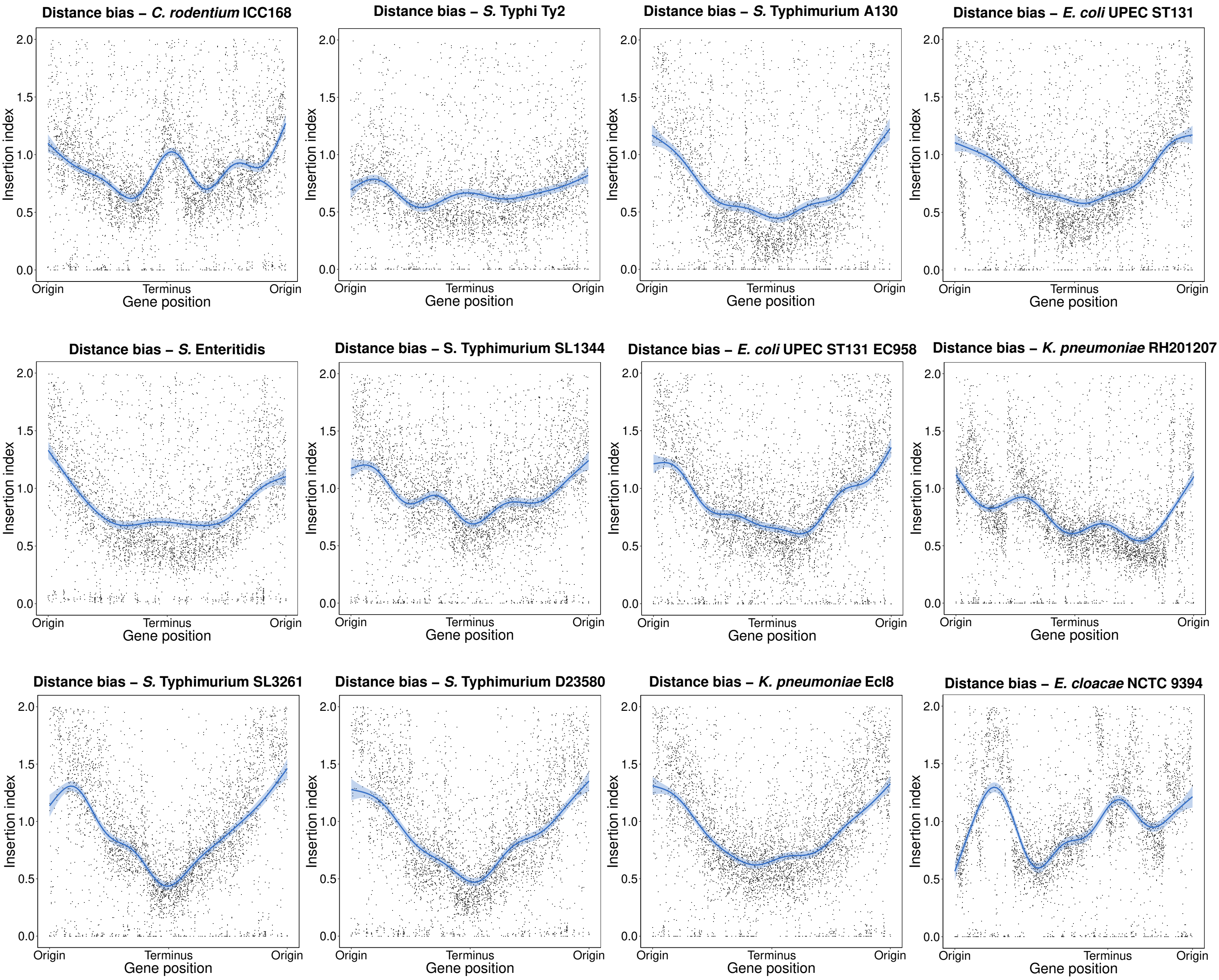

Figure S2: The effect of distance from the origin of replication on the insertion index. The origin of replication is assumed to be at the *dnaA* gene. Each point represents a gene and the blue line shows a GAM curve fit to the data with a 95% confidence interval. Some curves suggest possible scaffolding errors (e.g. *E. cloacae*). The plot for *E. coli* BW25113 is shown in the main text in Figure 3C.

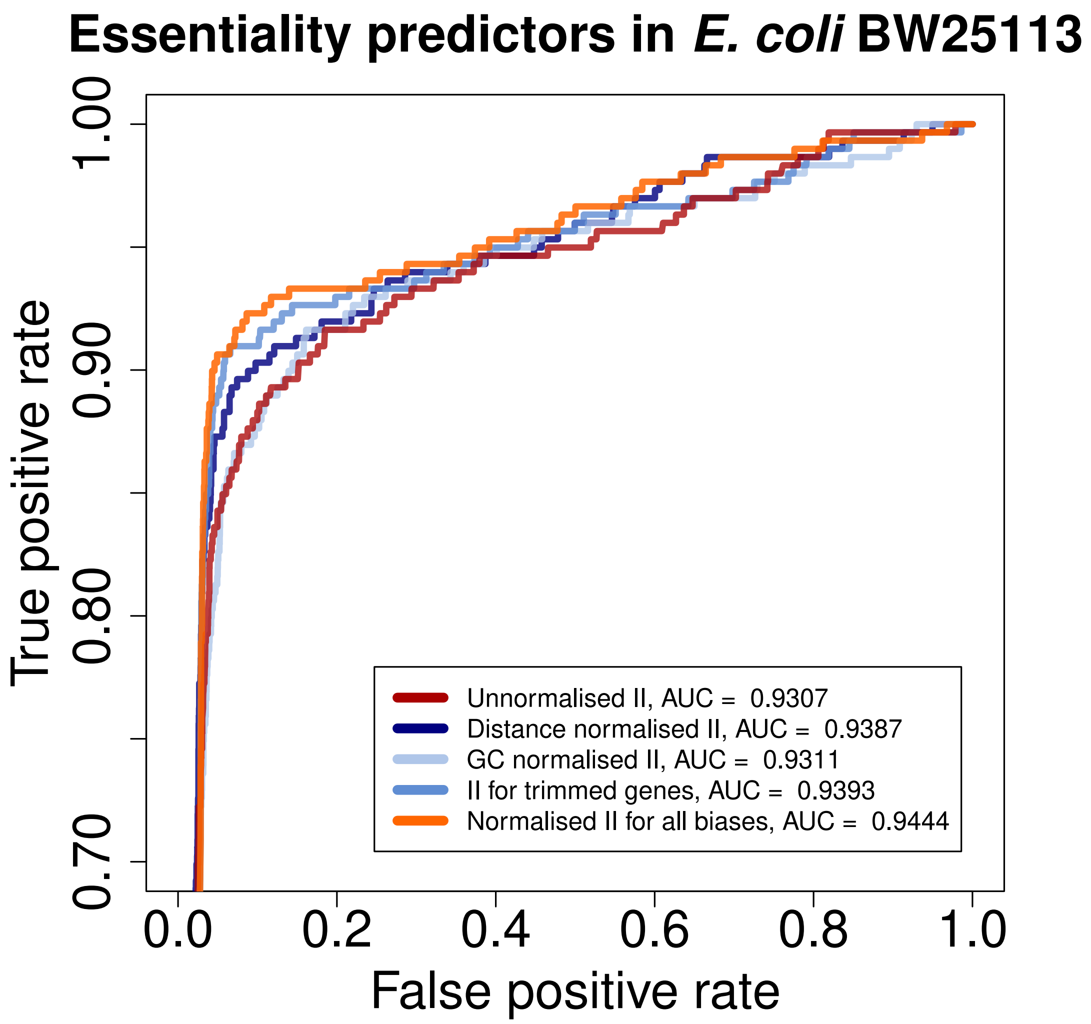

Figure S3: The effect of correcting various biases affecting the insertion index on the receiver operating characteristic (ROC) curve for predicting gene essentiality. As an example, normalizing for all biases increases the true positive rate from ~0.84 to ~0.9 at a false positive rate of ~0.05.

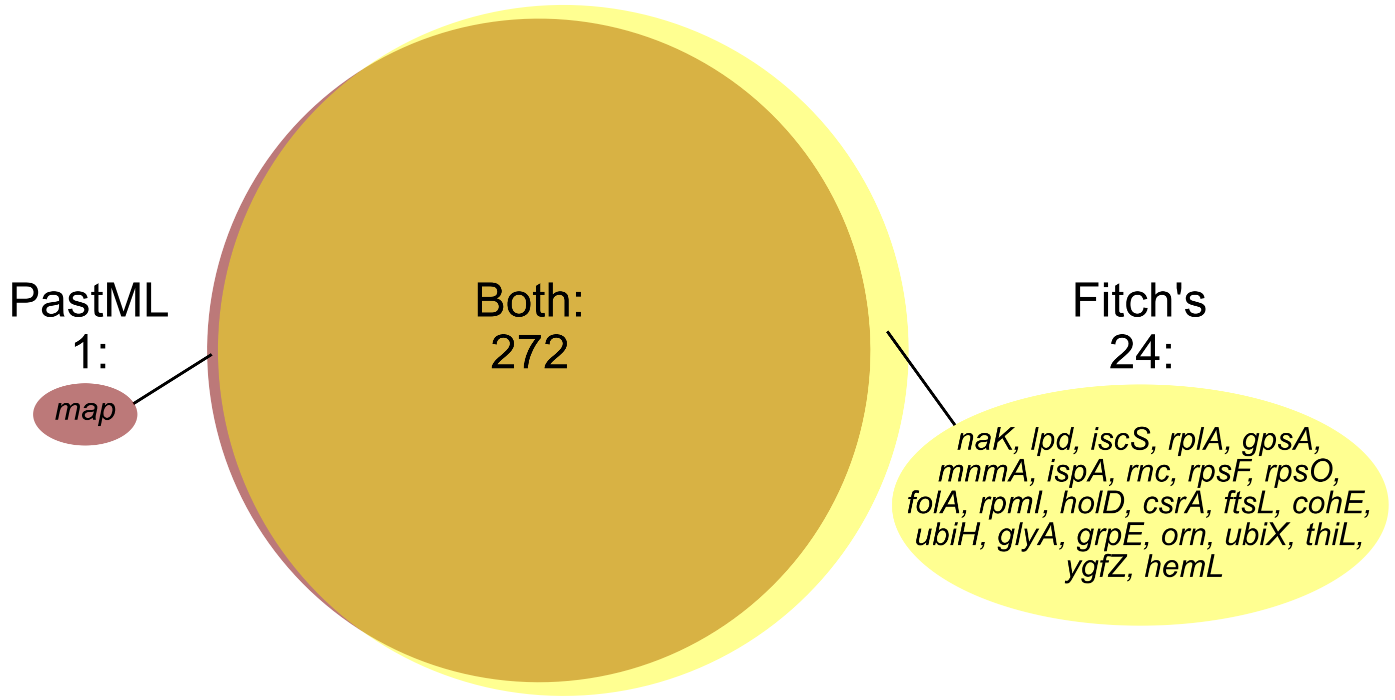

Figure S4: Euler plot showing the overlap of ancestrally essential genes called using either the PastML algorithm (left, red) or the Fitch's algorithm (right, yellow). Uniquely ancestral genes are shown in the bubbles with accordingly colored backgrounds.

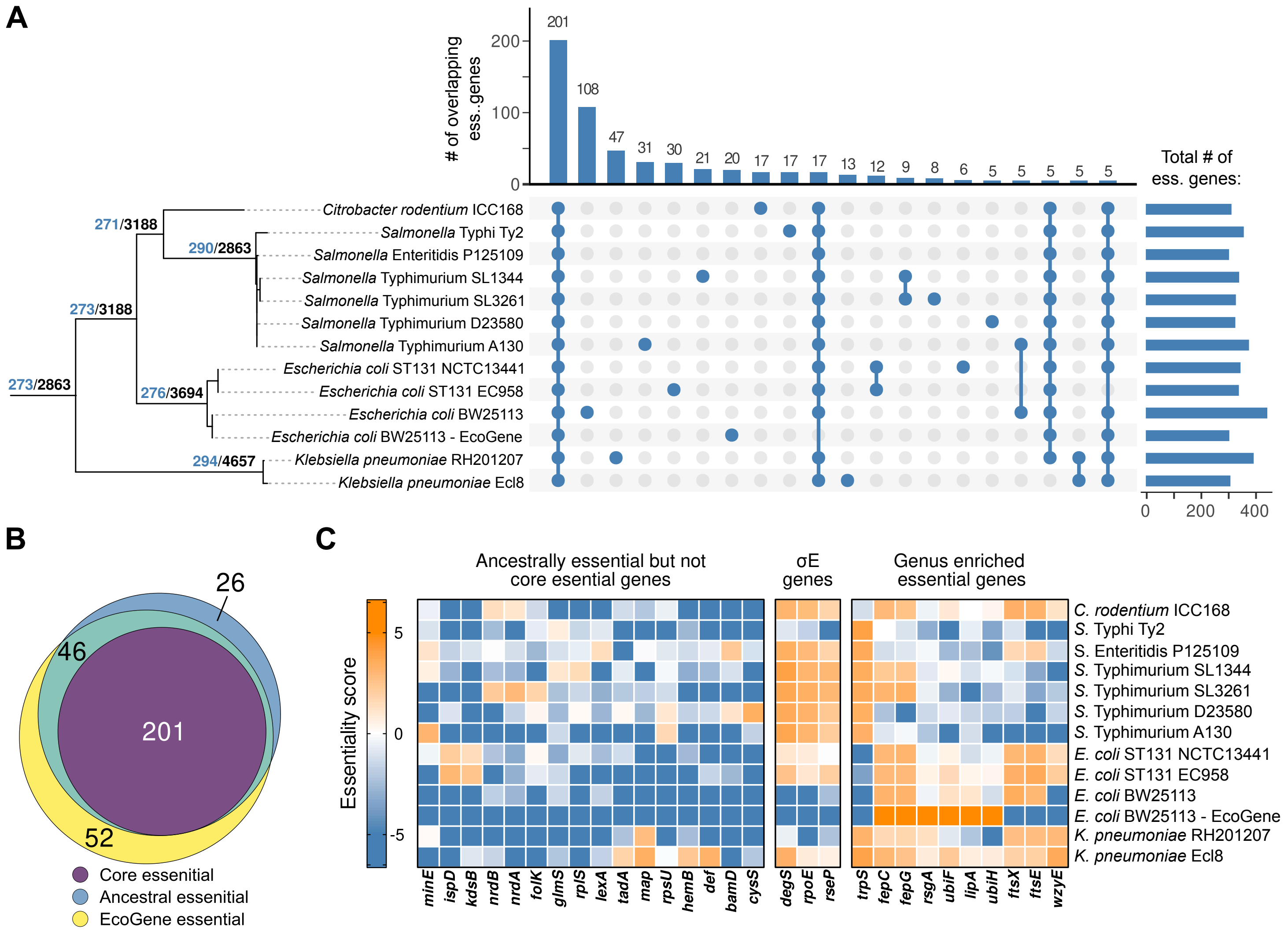

Figure S5: Reproduction of Figure 4 using the PastML algorithm for defining ancestral essentiality.

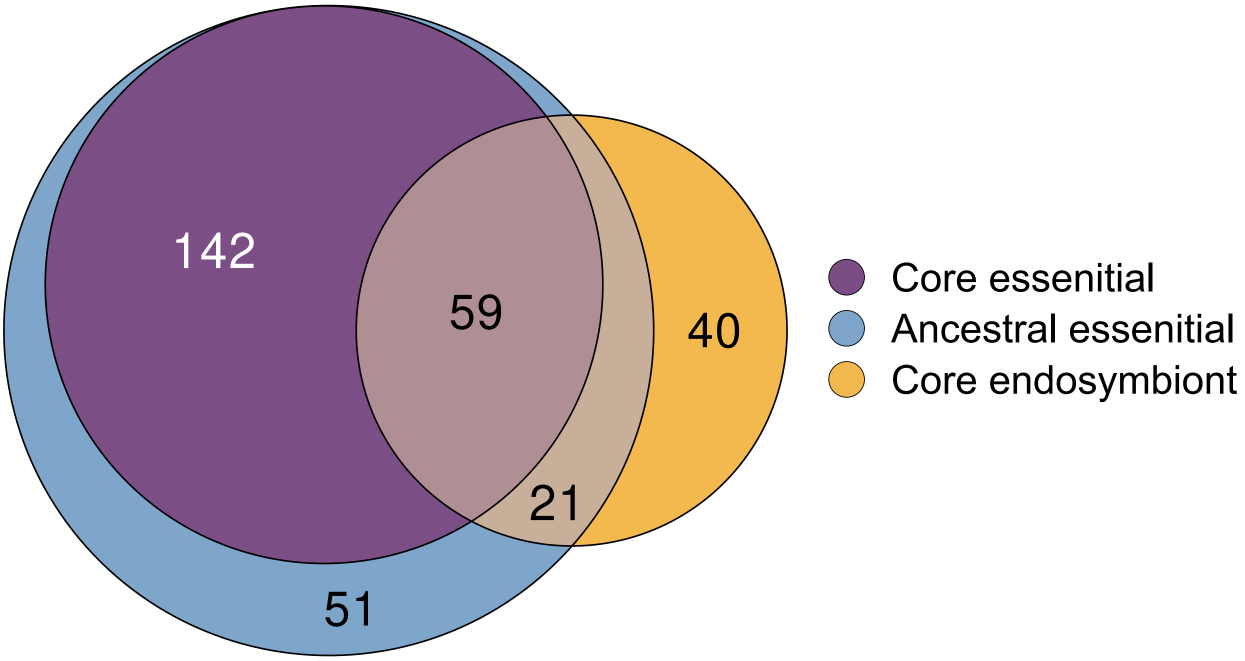

Figure S6: Reproduction of Figure 5 using the PastML algorithm for defining ancestral essentiality.

Table S1: Number of false negative calls at a set False positive rate of 0.05 in the ROC curves. Tot-FN denotes the number of genes that are expected to be false negatives (essential genes called nonessential) when using EcoGene essential genes as the gold standard.

Strain	Method	Tot-FN
K. pneumoniae Ecl8	Insertion index	28
K. pneumoniae Ecl8	PCA	26
E. cloacae NCTC 9394	Insertion index	21
E. cloacae NCTC 9394	PCA	21
C. rodentium ICC168	Insertion index	21
C. rodentium ICC168	PCA	18
S. Typhimurium SL1344	Insertion index	16
S. Typhimurium SL1344	PCA	15
S. Typhimurium D23580	Insertion index	22
S. Typhimurium D23580	PCA	21
S. Typhi Ty2	Insertion index	14
S. Typhi Ty2	PCA	17
K. pneumoniae RH201207	Insertion index	22
K. pneumoniae RH201207	PCA	21
E. coli UPEC ST131	Insertion index	25
E. coli UPEC ST131	PCA	23
S. Enteritidis	Insertion index	22
S. Enteritidis	PCA	20
S. Typhimurium SL3261	Insertion index	24
S. Typhimurium SL3261	PCA	23
S. Typhimurium A130	Insertion index	22
S. Typhimurium A130	PCA	20
E. coli UPEC ST131 EC958	Insertion index	30
E. coli UPEC ST131 EC958	PCA	29
E. coli BW25113	Insertion index	33
E. coli BW25113	PCA	30
